# Dataset for cognition processes, motivations, spatial presence experience, and customer engagement in retail mobile apps

**DOI:** 10.1016/j.dib.2022.108198

**Published:** 2022-04-28

**Authors:** Angelina Nhat Hanh Le, Huong Xuan Ho, Dong Phong Nguyen, Julian Ming Sung Cheng

**Affiliations:** a School of Management, University of Economics Ho Chi Minh City, 59C Nguyen Dinh Chieu, Ho Chi Minh City, Vietnam; b School of International Business and Marketing, University of Economics Ho Chi Minh City, 59C Nguyen Dinh Chieu, Ho Chi Minh City, Vietnam; c Business Administration Department, National Central University, No. 300, Chung-Ta Rd., Chung Li District, Taoyuan City, 32001, Taiwan, ROC

**Keywords:** Customer engagement, Spatial presence experience, Interactivity, Vividness, Need for cognition, Domain-specific interest, Hierarchy-of-effects model, Situated cognition theory

## Abstract

This article presents data for the estimation of a theory-driven dynamic and contingent model of customer engagement in the context of retail mobile apps. The data were collected from 558 participants who have installed at least one retail mobile app for a minimum of six months and have made relatively frequent purchases using the app. Customer-related data include participants’ interactivity and vividness cognitions, spatial presence experience, and engagement behaviors (i.e., customer purchases, referrals, influences, and feedbacks/suggestions) toward retail mobile apps. The data additionally include individuals’ tendency/motivation-related variables, such as need for cognition and domain-specific interest, which modulate customers’ cognitions as well as affective evaluations that are then followed by their actions. The authors collected the data from early May through mid-July 2020 in three major cities (i.e., Hanoi, Danang, and Ho-Chi-Minh) with leading positions in the Vietnamese eBusiness index. The presented data can be used to investigate the contingency model of driving factors of customer engagement in the context of retail mobile apps and improve the design and functionalities of mobile apps that foster embodied and embedded cognitions, facilitate the feeling of a “real” shopping experience, and ultimately encourage customers to actively engage and effectively contribute to participating retailers. For findings, discussions and further information, please refer to our recent research article: “Customer engagement in the context of retail mobile apps: A contingency model integrating spatial presence experience and its drivers” [1].

## Specifications Table

**Table:** 

Subject	Marketing
Specific subject area	Consumers’ interactive and vividness cognitions, need for cognition, domain-specific interest, spatial presence experience, and a variety of engagement behaviors with retailers through mobile apps
Type of data	.xlsx-fileTable
How the data were acquired	The research questionnaire was developed based on prior studies in the extant literature. Using the self-administrated survey, the dataset was collected from three major cities of Vietnam, namely Hanoi, Danang, and Ho-Chi-Minh. The survey form was provided as a supplementary file.
Data format	RawAnalyzed
Description of data collection	Data collection was conducted during a two-month period from early May through mid-July 2020. The targeted survey participants were comprised of mobile app shoppers who were residents of the three stated major cities. A quota sampling approach was carried out, taking into consideration residential status and age quota, to ensure an adequate representation of population characteristics. The participants in our inquiry were consumers who had installed at least one retail mobile app for a minimum of six months and had made relatively frequent purchases using the app. A total of 580 questionnaires were collected. Out of these, 558 valid responses were identified.
Data source location	• Institution: University of Economics Ho Chi Minh City• City/Town/Region: Ho-Chi-Minh City• Country: Vietnam• Latitude and longitude for collected samples/data: 14.058324, 108.277199
Data accessibility	Repository name: Mendeley DataData identification number: DOI: 10.17632/r96cfpkh82.2Direct URL to data: https://data.mendeley.com/datasets/r96cfpkh82/2
Related research article	H.X. Ho, P.D. Nguyen, J.M.S Cheng, A.N.H. Le, Customer engagement in the context of retail mobile apps: A contingency model integrating spatial presence experience and its drivers, J. Retailing Consum. Serv. 66 (2022) 102950. https://doi.org/10.1016/j.jretconser.2022.102950[1].

## Value of the Data

• The data presented are useful as they can contribute to a better understanding of the mechanisms from two mobile-app cognitions, namely interactivity and vividness, and their influence on customer engagement via spatial presence experience under the contingent roles of consumers’ personal tendencies and issue-specific motivations, which are scarce in customer engagement research. The reference results presented in [1] can be used to investigate new knowledge on drivers of customer engagement in the mobile-app environment.
• Researchers studying customer experience in technology-enabled services can benefit from these data through advancing knowledge pertaining to enablers that generate a specific experience, the feeling of “being” there [1], in virtual environments.
• The data presented are unique and provide insights into the concepts, namely interactivity, vividness, spatial presence experience, customer engagement, need for cognition, and domain-specific interest, which are still underexplored in the context of retail mobile apps. Therefore, these data and the analysis findings that are advocated in [1] allow for a better understanding of how spatial presence experience is elicited by situated cognitive processes and can stimulate consumers’ active engagement with retailers through mobile apps.
• Researchers can reuse these data to compare and contrast results from web/lab-based experiments and other survey studies in different contexts or cross-countries and compare the findings against their own.
• Practitioners can use these data to optimize personalized, authentic and realistic experiences within retail mobile apps through incorporating app design to ultimately boost customer engagement.
• The data can be used for insights into the centrality of the increasingly emphasized mode of online experience in the era of virtual environments augmented by immersively-enabled technologies. Researchers and managers alike are looking for new insights into the drivers of novel online experiences which then translate into customer engagement activities in the virtual world.

## 1 Data Description

This article contains data for an investigation of the intervening and conditional mechanisms from customer cognitions of technology-enabled functionalities to customer engagement through spatial presence experience in the context of retail mobile apps. The complete dataset obtained between early May and mid-July 2020 involves mobile app shoppers who have installed at least one retail mobile app for a minimum of six months and have made relatively frequent purchases using mobile apps in three Vietnamese cities leading the eBusiness index. The survey form included three main information sections: general information, measurement scales, and demographic-related information (see Supplementary file). Specifically, the first section consisted of the screening questions and available information related to respondents’ use and purchase experiences through retail mobile apps, including retailers’ mobile app installation and use (2 categories: yes and no), frequency of use (5 categories), purchase through mobile apps (2 categories: yes and no), frequency of purchase via mobile apps (5 categories), time installed and used mobile apps (4 categories), and the retailer's mobile app which is used the most frequently over the last six months (8 categories).

A quota-based sampling approach taking into consideration residential status and age was applied in attempting to obtain a sample that can represent the population. In this way, a total of 580 shoppers participated in and completed the survey. Responses that did not meet the requirements were excluded, leading to a final sample of 558 subjects. Of the 558 participants who completed usable surveys, 62.0% were located in Ho-Chi-Minh, 34.1% were young people from 18 to 25, 39.8% had incomes ranging from 5-9 million VND per month, 68.1% were female, 77.6% held an undergraduate degree, 52.9% had more than two years of experience using retail mobile apps, and weekly mobile-app usage frequency had the largest percentage with 33.3%.

Numerical data consisted of 61 items of four uni-dimensional and two multi-dimensional (second-order) constructs that were measured by a seven-point Likert scale (1 = strongly disagree to 7 = strongly agree). The reflective-reflective and reflective-formative hierarchical component models of the second-order constructs and the reflective measurements of the unidimensional constructs were provided in supplementary files. In particular, customer engagement, which is defined as the level of customer value contributions, both direct and indirect, to a specific retailer [1], was a reflective-reflective second-order construct that included four first-order reflective components: customer purchases (4 items), customer referrals (4 items), customer influences (4 items), and customer feedbacks (4 items). Interactivity, which is defined as “the degree to which users can interact with virtual contents or/and objects and can modify the format and content of the mediated environment” [1], was a reflective-formative second-order construct that consisted of three first-order reflective dimensions: active control (4 items), two-way communication (4 items), and synchronicity (4 items). In addition, the dataset consisted of items related to four reflective unidimensional constructs: vividness, spatial presence experience, need for cognition, and domain-specific interest. Specifically, vividness—the ability of technologies to produce a sensory-rich mediated environment [1]—included six items. Also, spatial presence experience, which is defined as the feeling of realism or “being there” [1], consisted of eight items. Besides, need for cognition—the personal tendency in which an individual enjoys thinking—consisted of eleven items. Finally, domain-specific interest, which refers to “an individual's motivational dispositions and the content or issue of a domain” [1], included eight items.

We validated the measurement scales of the components/constructs by performing reliability and validity analyses with the SmartPLS 3.3.2 software package [2]. Our measurement models included not only unidimensional constructs but also reflective-reflective (i.e., customer engagement) and reflective-formative (i.e., interactivity) types of second-order constructs; thus, as suggested by Sarstedt et al. [3], a disjoint two-stage approach of the sequential latent variable score method was employed to evaluate the measurement models. In Stage I, we computed item loadings, Cronbach's α, composite reliability (CR) values, and average variance extracted (AVE) values for assessing the reliability and convergence validity of the unidimensional constructs and the dimensions of the second-order constructs [4,5]. Table 1 presents that these reflective components/constructs had Cronbach's α and CR values greater than 0.70, thus the scales of all reflective components/constructs were considered reliable. Moreover, as shown in Table 1, several items were removed due to their standardized factor loadings being less than 0.50; the remaining index values exceeded the thresholds. In addition, AVE values were greater than 0.50, thus providing evidence for the convergence validity of these dimensions/constructs. We then deployed Fornell-Larcker's criterion, Heterotrait–Monotrait (HTMT) ratios, and factor structures [4,6] in order to evaluate the discriminant validity of these reflective components/constructs. The results indicated that the square root of AVE values for each dimension/construct were all greater than their largest correlations with other dimensions/constructs. Moreover, Table 2 shows that the HTMT ratios were significantly less than 0.85. In addition, we also found that the loading values on the corresponding dimensions/constructs were greater than their respective highest cross-loadings. A series of statistical tests thus provided evidence that the discriminant validity for all reflective dimensions/constructs was confirmed.

**Table 1:** Reflective scale accuracy analyses.

Reflective Components/Constructs and Items		Mean Value	Cronbach's Alpha	CRValue	AVE Value	Factor Loading
**Step I:** First-order reflective components (***bold italicized***) and unidimensional constructs (**bold**) were evaluated.						

***1. Active Control (ACT)***		***5.367***	***0.775***	***0.854***	***0.594***	
ACT1	I felt that I had a lot of control over my experience with the retail mobile app.					0.748
ACT2	While I used the retail mobile app, I could freely choose what I wanted to see.					0.747
ACT3	While I used the retail mobile app, I had absolutely no control over what I could do on the retail mobile app^R^.					0.811
ACT4	While I used the retail mobile app, my actions decided which kind of experiences I had.					0.775

***2. Two-way Communication (TWO)***		***4.875***	***0.793***	***0.866***	***0.617***	
TWO1	The retail mobile app facilitated two-way communication between the retailer and their consumers.					0.767
TWO2	The retail mobile app gave consumers the opportunity to talk to the retailer.					0.798
TWO3	The retail mobile app did not at all encourage visitors to talk back^R^.					0.810
TWO4	The retail mobile app made me feel the retailer wants to listen to their customers.					0.766

***3. Synchronicity (SYN)***		***5.038***	***0.816***	***0.878***	***0.644***	
SYN1	The retail mobile app processed my input very quickly.					0.727
SYN2	I was able to get information from the retail mobile app very rapidly.					0.783
SYN3	When I clicked on the retail mobile app, I felt I was getting the instantaneous information I expected.					0.816
SYN4	The retail mobile app was very slow in responding to my requests^R^.					0.878

**4. Vividness (VIV)**		**5.091**	**0.896**	**0.920**	**0.658**	
VIV1	The retail mobile app was very clear.					0.775
VIV2	The retail mobile app was very detailed.					0.821
VIV3	The retail mobile app was very vague^R^.					0.844
VIV4	The retail mobile app was very vivid.					0.800
VIV5	The retail mobile app was very sharp.					0.788
VIV6	The retail mobile app was very well-defined.					0.836

**5. Spatial Presence (SPAT)**		**4.770**	**0.848**	**0.885**	**0.524**	
SPAT1	I felt like the products were “actually there” in the retail mobile app.					0.698
SPAT2	It was as though the true location of the products had shifted into the retail mobile app.					0.736
SPAT3	I felt like the product meshed with the retail mobile app.					0.759
SPAT4	It seemed as if the products actually took part in the action in the retail mobile app.	Item removed during accuracy test				
SPAT5	I had the impression that I could be active with the products in the retail mobile app.					0.714
SPAT6	I felt like I could move the products around in the retail mobile app.					0.747
SPAT7	The products in the retail mobile app gave me the feeling I could do things with them.					0.735
SPAT8	It seemed to me that I could do whatever I wanted with the products in the retail mobile app.					0.674

***6. Customer Purchases (PUR)***		***4.770***	***0.864***	***0.908***	***0.712***	
PUR1	I would consider the retail mobile app as one of my first choices for buying products/services online.					0.795
PUR2	I will continue to buy products/services with the retail mobile app in the next few years.					0.869
PUR3	I would do more business with the retail mobile app in the next few years.					0.854
PUR4	My purchases with the retail mobile app make me content.					0.855

***7. Customer Referrals (REF)***		***5.306***	***0.842***	***0.894***	***0.678***	
REF1	I promote the retail mobile app because of the monetary referral benefits provided by the retailer.					0.802
REF2	In addition to the value derived from the products/services, the monetary referral incentives also encourage me to refer the retail mobile app to my friends and relatives.					0.835
REF3	I enjoy referring the retail mobile app to my friends and relatives because of the monetary referral incentives.					0.856
REF4	Given that I already use the retail mobile app, I refer my friends and relatives to the retail mobile app because of the monetary referral incentives.					0.800

***8. Customer Influences (INF)***		***4.335***	***0.879***	***0.917***	***0.734***	
INF1	I intend to share my ideas about the retail mobile app with other community users.					0.826
INF2	I love talking about my retail mobile app experience.					0.889
INF3	I discuss the benefits that I get from the retail mobile app with others.					0.855
INF4	I often mention the retail mobile app in my conversations.					0.856

***9. Customer Feedbacks (FEEB)***		***4.470***	***0.803***	***0.872***	***0.631***	
FEEB1	When I experience a problem with the retail mobile app, I provide feedback about my experiences to the retailer.					0.714
FEEB2	I provide suggestions/complete surveys for improving the performance of the retail mobile app.					0.772
FEEB3	I provide feedback/suggestions about the new facets of the retail mobile app.					0.858
FEEB4	I provide feedback/suggestions for developing new facets for the retail mobile app.					0.824

**10. Need for Cognition (NFC)**		**4.988**	**0.897**	**0.917**	**0.553**	
NFC1	I enjoy thinking of solutions to problems.					0.682
NFC2	I prefer solving a complex question that is difficult and requires thought, compared to a question that is important but does not require thought.	Item removed during accuracy test				
NFC3	I like situations where I can achieve something by thoroughly thinking things through.					0.712
NFC4	I love it when my life is full of difficult tasks that I have to solve.	Item removed during accuracy test				
NFC5	It is especially fun for me if I have completed an important task that requires a lot of thinking.					0.749
NFC6	I prefer complex problems over simple problems.					0.758
NFC7	I like to do tasks in which one has to think a great deal.					0.803
NFC8	I often say to myself that people should think long and carefully to find the best solution to a problem.					0.823
NFC9	I am someone who enjoys thinking.					0.801
NFC10	I like to think about a problem, even when I know that my thinking will change nothing about the problem.					0.772
NFC11	When I put my mind to solving a difficult problem, I usually succeed.					0.559

**11. Domain-Specific Interest (DSI)**		**4.747**	**0.912**	**0.931**	**0.694**	
DSI1	I am generally interested in the topic of retail mobile apps.					0.836
DSI2	Retail mobile apps corresponded very well with what I normally prefer.					0.837
DSI3	I have felt a strong affinity to the theme of retail mobile apps for a long time.					0.846
DSI4	There was already a fondness in me for the topic of retail mobile apps before I was exposed to them.					0.804
DSI5	Whenever I made a purchase, I would decide to deal with it via retail mobile apps.	Item removed during accuracy test				
DSI6	Things like those in retail mobile apps have often attracted my attention in the past.					0.857
DSI7	I just love to think about the topic of retail mobile apps.					0.818
DSI8	In the past, I have spent a lot of time dealing with the topic of retail mobile apps.	Item removed during accuracy test				

**Step II:** The second-order reflective construct is presented here while the second-order formative construct is shown in Table 5						

**12. Customer Engagement**			**0.763**	**0.851**	**0.591**	0.835
Customer Purchases						0.629
Customer Referrals						0.831
Customer Influences						0.762
Customer Feedbacks						

Notes:

R : Reverse-coded items; These values are based on a seven-point Likert-type scale ranging from “strongly disagree” (1) to “strongly agree” (7); CR: Composite Reliability; AVE: Average Variance Extracted. Interactivity is a reflective-formative second-order construct that includes: ACT, TWO, and SYN; Customer Engagement is a reflective-reflective second-order construct that includes: PUR, REF, INF, and FEEB.

**Table 2:** Discriminant validity assessment: Heterotrait-Monotrait (HTMT) ratios.

	Interactivity					Customer Engagement					
Constructs/Dimensions	Act	Two	Syn	Vividness	SPE	Pur	Ref	Inf	Fed	NFC	DSI
Interactivity*	n.a.			n.a.	n.a.	n.a.				n.a.	n.a.
Active Control (Act)	(**0.575**)										
Two-way Communication (Two)	0.525	(**0.702**)									
Synchronicity (Syn)	0.575	0.702	(**0.763**)								
**Vividness**	0.537	0.605	0.763	(**0.763**)							
**Spatial Presence Experience (SPE)**	0.459	0.628	0.652	0.707	(**0.776**)						
Customer Engagement*	n.a.			0.564	0.737	(**0.830**)				0.830	0.714
Customer Purchases (Pur)	0.350	0.497	0.529	0.464	0.586	(**0.736**)					
Customer Referrals (Ref)	0.517	0.415	0.512	0.490	0.487	0.482	(**0.593**)				
Customer Influences (Inf)	0.234	0.386	0.369	0.325	0.548	0.736	0.316	(**0.736**)			
Customer Feedbacks (Feb)	0.196	0.342	0.290	0.358	0.516	0.550	0.346	0.716	(**0.716**)		
**Need for Cognition (NFC)**	0.541	0.587	0.679	0.612	0.776	0.732	0.593	0.584	0.495	(**0.830**)	
**Domain-Specific Interest (DSI)**	0.263	0.391	0.371	0.417	0.510	0.539	0.498	0.504	0.526	0.471	(**0.714**)

**Notes:** Diagonal elements are the highest values of HTMT ratios and highlighted in bold and in parentheses.

⁎ Values for second-order constructs are obtained from Step II; n.a.: not applicable.

In Stage II, the latent variable scores of the dimensions obtained in Stage I were deployed as indicators/inputs for their corresponding second-order constructs. Consequently, customer engagement and interactivity developed into reflective and formative constructs, respectively; each of them was formed by the estimated latent variable scores of their corresponding dimensions. In Stage II, the scale reliability and validity of customer engagement were grounded on the same benchmarks as for the reflective scales in Stage I. In summary, the assessment values demonstrated an adequate level of reliability, convergent validity, and discriminant validity for customer engagement.

As mentioned earlier, interactivity was operationalized as a reflective-formative second-order construct. Thus, in Stage II, the latent scores of the three dimensions (i.e., active control, two-way communication, and synchronicity) obtained in Stage I were used as formative indicators for the second-order construct. The procedure to assess the validity of the formative scale of interactivity was adapted from prior studies (e.g., [3,7]). First, we calculated a “weighted” score for each dimension/indicator by multiplying its latent score by its PLS weight. The weighted scores were then summed to generate a composite score for the formative construct (i.e., interactivity). Subsequently, the three weighted scores were correlated against the composite score to produce dimension-to-construct correlations. The results in Table 3 indicate that all dimension-to-construct correlations were significant, thus providing evidence for convergence validity. Furthermore, the VIF values of active-control (1.324), two-way communication (1.545), and synchronicity (1.616) were far below the stringent threshold of 3.0 [5], suggesting that multicollinearity was not a threat to the formative measurement of interactivity. We, therefore, confirmed that the formative scale of interactivity was valid.

**Table 3:** Accuracy analysis of formative construct of interactivity.

**Formative construct**	**Indicators**	**Weight**	**Dimension-to-construct correlation**	**Highest cross-dimension correlation**	**Highest VIF**
Interactivity	Active Control	0.185⁎⁎	0.627⁎⁎⁎	0.415	1.324
	Two-way Communication	0.452⁎⁎⁎	0.846⁎⁎⁎	0.457	1.545
	Synchronicity	0.558⁎⁎⁎	0.899⁎⁎⁎	0.568	1.616

**Notes:** Significance:

⁎⁎⁎ p < 0.001.

⁎⁎ p < 0.010; VIF: Variance Inflation Factor.

## 2 Experimental Design, Materials and Methods

All the construct measurements were adapted from the validated scales of previous studies, with a slight modification to fit the retail mobile-app context. Specifically, the scale of interactivity which was operationalized as a reflective-formative second-order construct of three dimensions—active control, two-way communication, and synchronicity—with twelve items in total was adapted from Liu [8]. Vividness consisted of a six-item scale adapted from Yim et al. [9]. Using Hilken et al.’s [10] scale, spatial presence experience was measured as comprised of eight items. The scales of need for cognition and domain-specific interests were drawn from the eleven- and eight-item indexes of Vanwesenbeeck et al. [11] and Hartmann et al. [12], respectively. In addition, the measurement for customer engagement was derived from Kumar and Pansari's [13] reflective-reflective second-order construct comprised of four dimensions—customer purchases, customer referrals, customer influences, and customer feedback—and included sixteen items in total.

Because the data were collected in Vietnam (where the official language is Vietnamese), and all the measurement scales were originated from prior literature published in English, the back-translation technique was applied to ensure that the Vietnamese version of the scale items accurately conveyed the corresponding original meaning. In particular, the authors translated the constructs’ items into Vietnamese, and another bilingual marketing scholar subsequently translated it back into English. The Vietnamese version of the questionnaire was then carefully reconsidered and modified to better suit Vietnamese respondents. An in-depth discussion was held with three marketing professors who had profound knowledge regarding mobile commerce. Further, a pre-test with nine consumers who had experience with retail mobile apps was conducted to ensure there were no unambiguous phrases or illogical flows in the questionnaire. Based on their comments and suggestions, the final questionnaire was refined and used for data collection.

The survey form commenced with an introduction and an assurance of confidentiality of participant responses. The body of the survey form was structured into three sections. In order to ensure eligible responses, the first section included two screening questions that were used to verify if participants had installed at least one retail mobile app for a minimum of six months and had made relatively frequent purchases using retail mobile apps. Also, questions regarding the frequency of retail mobile app usage and frequency of purchases through retail mobile apps were asked to better understand the extent to which respondents had experience with online service consumption contexts. In addition, in this part, we also asked participants about the retail mobile app with which they shopped the most frequently. The second section then included all the statements measuring the studied constructs (i.e., interactivity, vividness, spatial presence experience, customer engagement, need for cognition and domain-specific interest) in the proposed theoretical model. Moreover, to limit the potential for the agreement bias tendency and recognize unengaged responses in the design of the survey instrument, we randomly interspersed reverse scale items throughout this section. In the last part of the questionnaire, questions were included for collecting information about respondents’ demographics such as residential status, gender, age, education, occupation, and income. Instructions in each section and part of the survey form were included to guide respondents on how to complete the questionnaire.

The intensive data collection was carried out in Vietnam from early May through mid-July 2020. The targeted survey participants were comprised of retail mobile app shoppers who were residents of three major cities—i.e., Hanoi, Danang, and Ho-Chi-Minh—occupying leading positions on the Vietnamese eBusiness index. A quota sampling technique, which can assure good representation with regard to target population characteristics [14], was applied to determine a sample in proportion to certain traits within the population. Consequently, the residential status and age quota were used to ensure an adequate representation of the target population. In each city, two well-trained interviewers were recruited and assigned to various supermarkets in main metropolitan districts to collect data. Interviewers intercepted people who had free time in the supermarkets and invited them to take part in filling the questionnaire out voluntarily. Two screening questions were asked to verify whether the participants had installed at least one retailer's mobile app for a minimum of six months and frequently purchased through the app, thus ensuring the eligibility of the respondents. A total of 580 questionnaires were distributed; after the data cleaning, a final sample of 558 questionnaires was identified as usable for data analysis. All data were processed through SPSS 26.0 and SmartPLS 3.3.2.

## Ethics statements

The study has followed all the established standards and was approved by the Ethics Committee of the University of Economics Ho Chi Minh City (No: 3822/QĐ-ĐHKTQLKH). The authors received informed consent from all participants. Their participation was voluntary and they could withdraw from the study at any point. As an ethical research team, we value the privacy rights of human subjects. Therefore, the identifiable information from the participants, such as names, addresses, and other personal/organizational details, are not captured in the survey to remain confidential and private. In addition, the data we submitted have not been acquired in violation of applicable law or by using human or animal subjects.

## CRediT Author Statement

**Angelina Nhat Hanh Le:** Funding acquisition, Project administration, Supervision, Conceptualization, Data curation, Validation csi103391, Writing - original draft, Writing - reviewing & editing; **Huong Xuan Ho:** Conceptualization, Data curation, Formal analysis, Investigation, Methodology, Writing - original draft, Writing - reviewing & editing; **Phong Dong Nguyen:** Funding acquisition, Supervision Validation csi103391, Writing - reviewing & editing; **Julian Ming Sung Cheng:** Funding acquisition, Conceptualization, Supervision, Validation csi103391, Writing - reviewing & editing.

## Declaration of Competing Interest

The authors declare that they have no known competing financial interests or personal relationships that could have appeared to influence the work reported in this paper.

## Data Availability

Dataset for cognition processes, motivations, spatial presence experience, and customer engagement in retail mobile apps (Original data) (Mendeley Data)
